# Gut microbiota–dependent Trimethylamine N-Oxide are related with hip fracture in postmenopausal women: a matched case-control study

**DOI:** 10.18632/aging.103283

**Published:** 2020-06-01

**Authors:** Yakun Liu, Yan-Long Guo, Shan Meng, Hua Gao, Li-Juan Sui, Shaobin Jin, Yang Li, Shao-Guang Fan

**Affiliations:** 1 Department of Pediatric Surgery, Qilu Hospital of Shandong University, Jinan, China; 2 Institute of Traumatic Orthopedics, The 80th Army Hospital of the Chinese People's Liberation Army, Weifang, Shandong Province, China; 3 Department of Rheumatology, Affiliated Hospital of Weifang Medical College, Weifang, Shandong Province, China

**Keywords:** Trimethylamine N-Oxide, hip fracture, postmenopausal women, Chinese

## Abstract

The study evaluates the serum levels of Trimethylamine N-Oxide (TMAO), a gut microbial metabolite, in 286 postmenopausal women with hip fracture. From January 1, 2018 to December 31, 2018, eligible patients were included. Same women without fracture mated age were enrolled. TMAO serum levels were tested by ultra-high-performance liquid chromatography-tandem mass spectrometry (UHPLC-MS/MS). The serum levels of TMAO were significantly higher in patients with hip fracture than in those controls (P<0.001). The serum levels of TMAO were also higher in patients with hip fracture only than in those who also had upper limb fracture (P=0.001). High level of TMAO was proved a predictor of both hip fracture and had upper limb fracture combined hip fracture, after the adjustment of other existing risk factors [e.g., for each 1 uM increase of TMAO, odd ratio 1.16 (95% CI, 1.07–1.25), P < 0.001; and 1.12 (95% CI, 1.03–1.26), P=0.008, respectively]. In summary, increased TMAO serum levels associated with high risk of hip fracture, suggesting that increase TMAO may contribute to osteoporosis and fracture in postmenopausal women.

## INTRODUCTION

The incidences of osteoporosis and fractures is rising rapidly due to the aging society in China [1]. Age is an independent risk factor for fracture and global aging brings urgency to fracture prevention [2]. Abnormal gut microbiota can affect the health of the elderly [3]. Previous studies showed that gut microbiota affected both digestive system and immune system, and associated with the osteoporosis and fractures [1]. The microbes are independent risk factors for obesity, diabetes, and osteoporosis [4].

Trimethylamine-N-oxide (TMAO), a circulating organic compound, derived from the metabolism of dietary L-carnitine and choline (metabolized by intestinal bacteria trimethylamine) [5]. Previous study had suggested that TMAO were associated with diabetes [6], metabolic syndrome [7], hypertension [8], stroke [9] and cardiovascular diseases [10].

Li et al. [11] reported that the rate of lose bone starting in midlife was ranged from 0.3% to 0.5% per year. The gut microbiota can affect bone density, bone remodeling and bone health [12–13]. However, the relationship between TMAO and fractures in Chinese patients has not been previously reviewed. We suspected that serum levels of TAMO were associated with osteoporosis. Furthermore, osteoporosis is the main cause of hip fractures [2]. Therefore, the study evaluates the serum levels of Trimethylamine N-Oxide (TMAO), a gut microbial metabolite, in 286 postmenopausal women with hip fracture.

## RESULTS

### Patient characteristics

From the initial 339 hip fracture postmenopausal women, 286 were included (53 patients were excluded: 28 with a major trauma or cancer; 5 with gastrointestinal diseases; 4 with infection and/or use of antibiotics; 6 with impaired hepatic/ renal function; 5 death and 5 referrals during research). The median age of those women was 65 (IQR, 57–75) years. 47.3% of the patients had associated comorbid illnesses, 15.4% had more than 1 comorbid illnesses, and the rest had a single comorbid illness. In addition, 11.5% (33/286) of the included patients had a family history of fragility fractures. Table 1 presents the baseline characteristics of the included women.

**Table 1:** Baseline characteristics of patients with hip fracture and controls^†^.

**Parameters**	**Patients**	**Controls**	**P**
N	286	286	
Age (years)	65(57-75)	65(57-75)	1.00
Han, n (%)	264(92.3)	264(92.3)	1.00
BMI(Kg/m2)	26.2(23.9-28.6)	25.9(23.7-27.9)	0.92
Drinking, n (%)	31(10.8)	27(9.4)	0.58
Smoker, n (%)	42(14.7)	25(9.7)	0.03
History of falls during the last year	1(1-2)	0(0-1)	0.009
Hip fracture type, n (%)		—	
Femoral head fracture	134(46.9)		
Femoral neck fracture	113(39.5)		
Subtrochanteric	39(13.6)		
Concomitant upper-limb fractures, n (%)	25(8.7)	—	
Distal radius (%)	15(5.2)	—	
Proximal humerus (%)	10(3.5)	—	
Total hip BMD(g/cm2)	0.68(0.63-0.70)	0.75(0.73-0.79)	<0.001
Patients had associated comorbid illnesses, n (%)	125(47.3)	115(40.2)	0.40
Diabetes	35(12.2)	27(9.4)	0.28
Hypertension	59(20.6)	50(17.5)	0.34
Cardiovascular disease	42(14.7)	25(8.7)	0.03
Hyperlipoproteinemia	33(11.5)	35(12.2)	0.80
Time between fracture and blood collection(hs)	22.5(15.0-28.5)	—	
Hospital stay(days)	8(4-12)	—	
Laboratory findings			
Serum calcium (mmol/l)	2.35(2.25-2.46)	2.44(2.34-2.65)	0.02
Serum ALP (IU/l)	232(150-340)	155(100-230)	<0.001
Serum Hs-CRP (mg dL^-1^)	0.35(0.24-0.45)	0.28(0.18-0.37)	0.007
Serum TMAO (uM)	5.8(3.4-8.8)	4.3(2.6-6.4)	<0.0001

^†^Results are expressed as N(percentages) or as medians (IQR).

NS, no significant; BMI, body mass index; Hs-CRP, High-sensitivity- C-reactive protein (BMI); ALP, alkaline phosphatase; TMAO, Trimethylamine-N-oxide; BMD, bone mineral density.

### Main results

**Figure 1:**
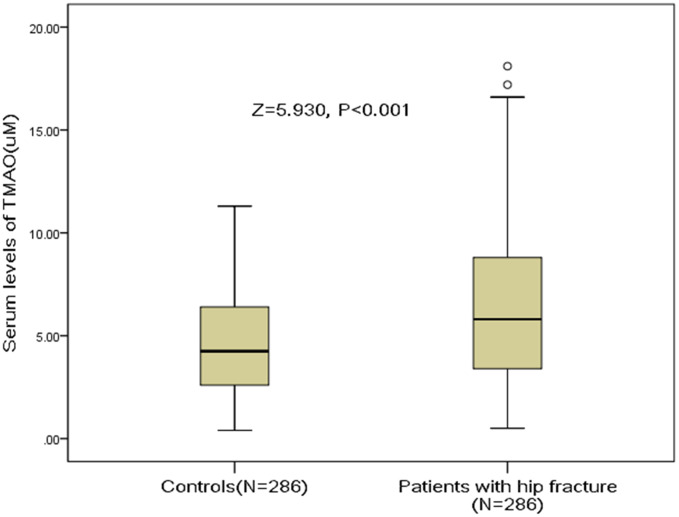
**Distribution of serum levels of TMAO in postmenopausal women with hip fractures and in controls.** All data are medians and inter-quartile ranges (IQR). P values refer to Mann-Whitney U tests for differences between groups. TMAO= Trimethylamine-N-oxide.

The median serum levels of TMAO in hip fracture patients was 5.8(IQR, 3.4-8.8) uM, which was significantly (P<0.001) higher than in those controls [4.3 (IQR, 2.6–6.4) uM], Figure 1. A negative correlation between TMAO and BMD (r=0.183, P=0.004) was found. The TMAO serum levels also related to high-sensitivity C-reactive protein (r=0.212, P<0.001) and Ca (r=0.186, P=0.003) in hip fracture patients. The serum levels of TAMO were associated with the number of falls in hip fracture patients (r=0.439, P<0.001) and in controls (r=0.338, P<0.001). No correlations were found between the serum levels of TMAO and the remaining binary variables (all, P>0.05).

### TMAO and risk of hip fracture

The OR of TMAO levels compared with other risk factors was calculated by the logistic regression analysis. High level of TMAO was a predictor of hip fracture [e.g., for each 1 uM increase of TMAO, the unadjusted OR and adjusted OR were 1.25[95% CI: 1.15–1.34] and 1.16 [1.07–1.25], respectively. As shown in the Table 2, smoker, history of falls during the last year, cardiovascular disease, serum levels of calcium, ALP and Hs-CRP were also the fracture predictors.

**Table 2:** Multivariable conditional logistic regression analysis for hip fracture^†^.

**Parameter**	**OR^‡^**	**95% CI**	***P***
Smoker (Yes vs. no)	1.07	1.02-1.22	0.04
History of falls during the last year	1.67	1.13-2.58	0.02
Cardiovascular disease (Yes vs. no)	1.15	1.03-1.35	0.03
Serum calcium	1.43	1.21-1.65	0.01
Serum ALP	1.03	1.01-1.06	0.009
Serum Hs-CRP	1.11	1.04-1.22	0.006
Serum TMAO	1.16	1.07-1.25	<0.001

^†^Multivariable model included all of the following variables: smoker, history of falls during the last year, cardiovascular disease, serum levels of calcium, ALP, hS-CRP and TMAO which had been confirmed in the univariable logistic regression analysis

^‡^Note that the odds ratio corresponds to a unit increase in the explanatory variable.

**Figure 2:**
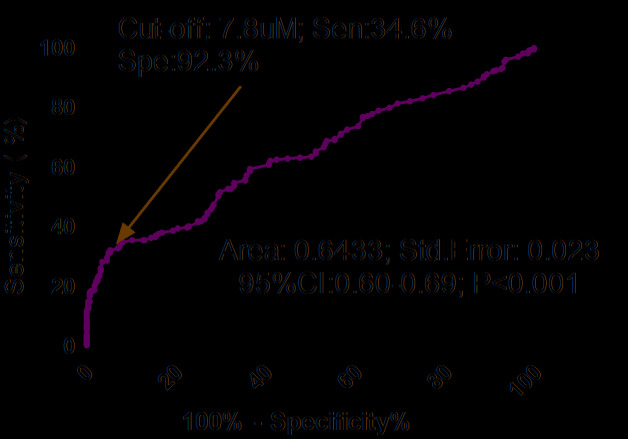
**Receiver operating characteristic (ROC) curves were utilized to evaluate the accuracy of serum TMAO levels to predict hip fractures.** TMAO= Trimethylamine-N-oxide.

As shown in the Figure 2, the optimal cutoff level of serum TMAO was estimated to be 7.8uM, which yielded an AUC of 0.64 (95% CI, 0.60–0.69), and the sensitivity and specificity were 34.6% and 92.3%, respectively. Similarly, multivariable analysis models were used to assess adjusted OR and 95% CI of hip fracture for elevated TMAO (defined as TMAO≥cut-off value; with normal TMAO as reference). The results showed that elevated TMAO related to high risk of hip fracture (unadjusted OR 6.38, 95% CI: 3.84–10.59). In multivariable analysis, there was an increased risk of hip fracture in those patients with elevated TMAO (OR 3.68, 95% CI: 2.04–5.96; P<0.001) after adjusting for above confounders.

### TMAO and risk of hip and upper limb fracture

**Figure 3:**
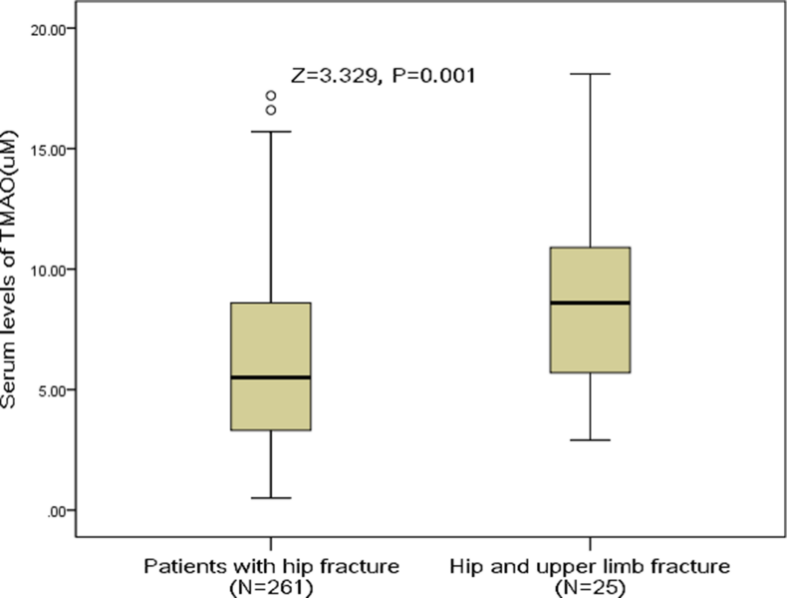
**Distribution of serum levels of TMAO in postmenopausal women with hip fractures only and with hip combined upper limb fractures.** All data are medians and inter-quartile ranges (IQR). P values refer to Mann-Whitney U tests for differences between groups. TMAO= Trimethylamine-N-oxide.

Twenty-five patients had concomitant upper limb fracture. The median serum levels of TMAO in those patients was 8.6(IQR, 5.5-11.2) uM, which was significantly (P<0.001) higher than in those patients with hip fracture only [5.5 (IQR, 3.3–8.6) uM], Figure 3. The OR of TMAO levels compared with other risk factors was calculated by the logistic regression analysis. High level of TMAO was a predictor of hip and upper limb fracture [e.g., for each 1 uM increase of TMAO, the unadjusted OR and adjusted OR were 1.20[95% CI: 1.08–1.34] and 1.12 [1.03–1.26], respectively.

**Figure 4:**
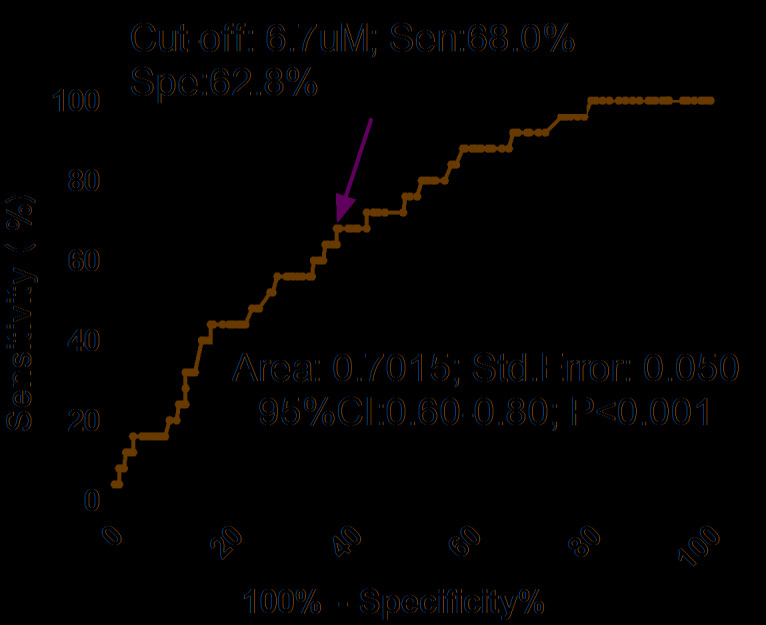
**Receiver operating characteristic (ROC) curves were utilized to evaluate the accuracy of serum TMAO levels to predict hip and upper limb fractures.** TMAO= Trimethylamine-N-oxide.

As shown in the Figure 4, the optimal cutoff level of serum TMAO was estimated to be 6.7uM, which yielded an AUC of 0.70 (95% CI, 0.60–0.80), and the sensitivity and specificity were 68.0% and 62.8%, respectively. Similarly, multivariable analysis models were also used to calculate adjusted OR and 95% CI of hip and upper limb fracture for elevated TMAO (defined as TMAO≥cut-off value; with normal TMAO as reference). The results showed that an increased risk of hip and upper limb fracture was associated with elevated TMAO (unadjusted OR 3.01, 95% CI: 1.28–7.06; P=0.012). In multivariable analysis, there was an increased risk of hip and upper limb fracture in those patients with elevated TMAO (OR 1.75, 95% CI: 1.21–2.43; P=0.039) after adjusting for possible confounders.

## DISCUSSION

TMAO, a gut microbial metabolite, plays role in osteoporosis and fractures [12, 14–15]. In this study, we measured TMAO serum levels of in hip fractures postmenopausal women and age matched healthy women and further assessed its association with fractures risk. The findings were: (1) TMAO serum levels were higher in hip fractures patients than in normal controls; (2) high TMAO levels related to low OR, odds ratio; CI, confidence interval; Hs-CRP, High-sensitivity- C-reactive protein; ALP, alkaline phosphatase; TMAO, Trimethylamine-N-oxide

BMD levels; (3) High level of TMAO was proved a predictor of both hip fracture and had upper limb fracture combined hip fracture, after the adjustment of other existing risk factors [e.g., for each 1 uM increase of TMAO, odd ratio 1.16 (95% CI, 1.07–1.25), P < 0.001; and 1.12(95% CI, 1.03–1.26), P=0.008, respectively].

Bone homeostasis is affected by Gut microbiota composition and/or products. Gut microbiota plays role in the various determinants of bone health. Xu et al. [14] reported that bone loss in postmenopausal osteoporosis, which is influenced by the intestinal microbiota, was associated with host immunity [14]. Another study showed that gut microbiota was a regulator of bone mass and it could effect the gut microbiota on bone mass through the immune system, which in turn regulates osteoclastogenesis [15]. Another study indicated that gut microbiota was an important factor in osteoporosis development [12].

In this study, we reported that gut microbiota–dependent TMAO was associated with hip fracture in postmenopausal women. Similarly, Black et al. [16] showed that uremic toxins from the gut (e.g., p-cresyl sulfate and indoxyl sulfate) might be involved in the development of bone disease in chronic kidney disease patients and Sjögren et al. [17] also showed that the gut microbiota was a risk factors for bone mass in mice.

Elevated serum levels of TMAO might play role in concomitant fractures. However, the biological mechanism between TMAO and fracture is still uncertainty. There are some possible mechanisms that may involve. First, alterations in the gut microbiome are associated with inflammatory conditions, which related to bone loss through changes in the gut–immune–skeletal axis [18]. In the study, we also reported that serum levels of TMAO were associated with H-CRP. Second, gut microbiota could alter immune status in bone and thereby affected bone resorption [17]. The gut microbiome related to bone loss in several conditions through immune activation and chronic inflammation [18]. Third, the gut microbiome could influence bone condition by altering nutrient absorption, stimulating the immune system or accelerating the transport of microbial products across the gut endothelium [13]. Fourth, gut microbiome could increase calcium absorption and modulate the production of gut serotonin, which was a bone mass regulator [19]. Lastly, gut microbiota could be a regulator of bone mass through mediation of the immune system activation, intestinal calcium absorption and the release of neurotransmitters [12, 20]. Gut microbiota provided an anabolic stimulus to the skeleton, which was intervened by insulin-like growth factor 1 [21].

In this study, we firstly assessed the association between TMAO serum levels and risk of hip fracture in postmenopausal women. However, this observational study does not allow advancing any cause and effect relationships. Whether TMAO directly contributes to fracture in postmenopausal women or circumstances around the fracture have caused changes in the serum levels of TMAO could not be confirmed.

This study included some other limitations. (1) We only included Chinese women, which may affect the generalizability. (2) Hospitalization and/or acute stress associated with the fracture incident [22–23], would most probably change the gut microbiome very rapidly [24–25] and therefore represents a major confounder for this analysis. We should consider this factor in the further study. (3) TMAO levels were tested one time at admission, and the serum TMAO concentrations in male patients were not measured. In addition, patients with hip fracture displayed lower calcium and increased alkaline phosphatase, which might signal presence of more osteomalaica in the hip fracture cohort. As several previous studies have demonstrated much lower vitamin D levels in hip fracture patients [2, 26–27]. The relationship between the serum levels of TMAO, vitamin D and hip fracture need to be elaborated in future research. (4) How serum levels of TMAO are related to changes in gut microbiome are important. Does it reflect an inflammatory gut flora? However, in this study, we did not obtain that information. They need to elaborate more on this issue in further study. (5) The receiver operating characteristic curves are used in the evaluation of the accuracy of serum TMAO levels in predicting individual fracture occurrences, but the evaluation is neither based on internal nor external validation [28].

In summary, we explored serum levels of TMAO changes in Chinese postmenopausal women with hip fracture. Increased TMAO serum levels associated with high risk of hip fracture, suggesting that increase TMAO may contribute to osteoporosis and fracture in postmenopausal women. Further studies are needed to assess the TMAO as a novel therapeutic target for osteoporosis and fracture.

## MATERIALS AND METHODS

### Participants

This was a single-center cross-section study. The study period was from January 1, 2018 to December 31, 2018. All postmenopausal women with hip fracture from the Department of Orthopedics of the 89^th^ Hospital of People's Liberation Army (Weifang, China) were screened. The hip fracture was diagnosed by X-ray examination. Exclusion criteria included: (1) gastrointestinal diseases, metabolic syndrome and cancer; (2) infection and/or use of antibiotics; (3) fractures caused by a traffic accident; (4) bilateral hip arthroplasty, hypocalcemia, liver and kidney dysfunction; (5) using estrogens or other bone-active therapies; (6) death or referral during research.

For each patient, one healthy postmenopausal woman matched for age, ethnicities, and body mass index (BMI) was chosen as control case. These healthy women came from our hospital medical examination center. The median age of those women was 65 (IQR, 57–75) years. The exclusion criteria of the control group were consistent with the patient group.

### Clinical information

All included women need to finish a standardized questionnaire, which was used to assess the putative risk factors of fractures. The information about age, ethnicities, current tobacco or alcohol use(yes or no), BMI, basic illness (hypertension, diabetes mellitus, cardiovascular disease, neurological disorders, and hyperlipoproteinemia), falls during the last year(yes or no), hip fracture type (cervical or trochanteric), surgical procedure type (arthroplasty or internal fixation) and family history of fragility fractures were obtained. The included patents also were divided into two groups: a hip fracture group and a concomitant fracture of the hip and upper limb fracture group [11]. In addition, total hip bone mineral density (BMD) of the proximal femur was measured using DXA (Hologic QDR2000/QDR4500).

### TMAO measurement

At 8:00 am the next day after enrollment, the blood samples of enrollees were collected. Serum was separated and stored at -80°C until analysis. Serum levels of TMAO were tested by ultra-high-performance liquid chromatography-tandem mass spectrometry (UHPLC-MS/MS) [29]. The UHPLC-MS/MS system consisted of an Accela autosampler, accela UHPLC binary pump, and a TSQ Quantum Ultra triple quadruple mass spectrometer (Thermo Fisher Scientific, San Jose, CA). The intra-and inter-assay coefficients of variation (CV) were 4.5%-6.0% and 5.0%-8.0%, respectively. The assay described shows good reproducibility accuracy (>98.0%).

### Statistical analysis

Continuous variable was presented as median and interquartile ranges (IQRs]), while categorical variable was expressed as number (%). The differences between groups were tested by Chi-square (categorical variable) and Mann-Whitney U test (continuous variable) [30]. Bivariate correlation was evaluated using Spearman's rank correlation.

Conditional logistic regression analysis (univariable and multivariable) was used to assess the association between serum TMAO levels and risk of fracture. Multivariable model included the following variables: smoker, history of falls during the last year, cardiovascular disease, serum levels of calcium, alkaline phosphatase, high-sensitivity- C-reactive protein and TMAO which had been confirmed in the univariable analysis. The results were presented as odds ratios (OR) and 95% confidence intervals (CI).

Lastly, the accuracy of serum TMAO in predicting fracture was assessed by the receiver operating characteristic (ROC) curves and the area under the ROC curve (AUC) was presented as the results [31]. The optimal cutoff level of serum TMAO was determined. Logistic regression analysis was also used to calculate the OR (95% CI) of fracture for elevated TMAO (defined as TMAO≥cut-off value; with normal TMAO as reference). SPSS 24.0 (SPSS Inc., Chicago, IL, USA) and GraphPad Prism 5.0 (GraphPad Software, San Diego, CA, USA) were used to perform all statistical analyses and statistical significance was defined as P<0.05.

### Ethical statement

The ethics committee of the 89^th^ Hospital of People's Liberation Army approved this research protocol. Written informed consents were obtained from all participants.
